# Ginseng Compatibility Environment Attenuates Toxicity and Keeps Efficacy in Cor Pulmonale Treated by Fuzi Beimu Incompatibility Through the Coordinated Crosstalk of PKA and Epac Signaling Pathways

**DOI:** 10.3389/fphar.2018.00634

**Published:** 2018-06-15

**Authors:** Yingying Huang, Lili Li, Xiaojin Li, Simiao Fan, Pengwei Zhuang, Yanjun Zhang

**Affiliations:** ^1^Chinese Materia Medica College, Tianjin University of Traditional Chinese Medicine, Tianjin, China; ^2^Tianjin State Key Laboratory of Modern Chinese Medicine, Tianjin University of Traditional Chinese Medicine, Tianjin, China

**Keywords:** Ginseng, compatibility environment, cor pulmonale, signal crosstalk, attenuation synergia

## Abstract

Cor pulmonale is characterized by severe right ventricular dysfunction caused by lung disease, particularly chronic obstructive pulmonary disease, which can lead to pulmonary hypertension. Our previous study has demonstrated that Fuzi and Beimu compatibility (FBC), a traditional TCM compatibility taboo, improves lung function in early-stage of pulmonary hypertension through the synergistic action of β-ARs signals. However, FBC increases cardiotoxicity with prolonged treatment and disease progression. Considering that the compatibility environment influences the exertion of the medicine, we selected ginseng for coordinating the compatibility environment to improve the security and extend the therapeutic time window of FBC. Monocrotaline-induced cor pulmonale rats were treated with FBC, ginseng, or ginseng combined with FBC (G/FBC). Then, the pulmonary and cardiac functions of the rats were examined to evaluate the toxicity and efficacy of the treatments. The crosstalk between PKA and Epac pathways was also studied. Results showed that G/FBC ameliorated lung function similar to or even better than FBC treatment did. Furthermore, G/FBC treatment attenuated FBC-induced cardiotoxicity, which significantly restored cardiac dysfunction and clearly decreased myocardial enzymes and apoptosis. The βAR-Gs-PKA/CaMKII pathway was inhibited and the Epac1/ERK1/2 axis was activated in G/FBC group. These findings indicate that ginseng compatibility environment could improve pulmonary function and attenuate cardiotoxicity in cor pulmonale via the coordinated crosstalk of PKA and Epac pathways, implying that ginseng could be used to prevent detrimental cardiotoxicity in cor pulmonale treatment.

## Introduction

Cor pulmonale is defined as right ventricular failure secondary to pulmonary hypertension, which is primarily caused by various pulmonary diseases (e.g., COPD or pulmonary vascular disease). In cor pulmonale, the increased afterload imposed on the right ventricle (RV) generates a maladaptive response, i.e., an alteration in the structure and function of the RV(Han et al., 2007; Weitzenblum and Chaouat, 2009; Kawut et al., 2014). Early identification of the predisposing factors and pro-active preventative measures could trigger specific therapeutic strategies to preserve cardiac function. Recommendations in relation to chronic obstructive pulmonary disease (COPD) treatment includ protective mechanical ventilation, bronchodilators, corticosteroids, and/or antibiotics (Vogelmeier et al., 2017). With β_2_-adrenergic receptors (β_2_-ARs) being important targets in COPD therapy, β_2_-agonists and phosphodiesterase-4 inhibitors have bronchus-expanding efficacy in patients with COPD in clinical trials (Bremner et al., 2018). Our previous study has demonstrated that Beimu acts synergistically with Fuzi on β_2_-ARs activation, and that Fuzi and Beimu compatibility (FBC) could ameliorate lung function and alleviate pulmonary histopathological changes in early-stage of pulmonary hypertension. However, this advantage needs to be considered carefully in relation to the increased side effects associated with cardiotoxicity when β_2_-ARs are persistently stimulated during heart failure (Zhuang et al., 2016). These studies indicate that the therapy for cor pulmonale is complex.

Extensive studies have established that β-adrenergic signaling in heart failure demonstrates cardiotoxic and cardioprotective effects (Insel et al., 2012; Zhang et al., 2013; Baker, 2014). Cardiac β-ARs transduce the signal produced by catecholamine stimulation via Gs proteins to their downstream effectors to increase cardiac contractility. However, persistent β-ARs system activation during heart failure causes myocyte death and cardiac decompensation (Taira et al., 2008; Woo et al., 2014). β-AR-mediated increases in cAMP activates protein kinase A (PKA) or exchange protein activated by cAMP (Epac), which is a newly identified cAMP signaling target that is independent from PKA. Recent studies have proposed PKA as a target to enhance myocardial apoptosis by the PKA-Ca/CaMKII pathway; they also hypothesized that the mechanisms underlying the anti-apoptotic response to cAMP involve Epac-ERK1/2 signaling (Insel et al., 2012; Zhang et al., 2013).

Chinese medical science has long realized that drug compatibility environment shows a unique advantage in the treatment of chronic and complex diseases. Through a flexible combination, it could improve curative effects, expand the scope of treatment, reduce side effects, and even adapt to various pathological changes (Su et al., 2016). The proper adjustment of drug combination could strengthen curative effect. Ginseng, a popular herbal medicine, is often used in combination with other drugs to achieve the maximum therapeutic response (Qian et al., 2017; Zhao et al., 2017).

The present study hypothesizes that ginseng combined with FBC (G/FBC) not only alleviates pulmonary function but also attenuates cardiotoxicity caused by FBC persistently stimulated β_2_-ARs. This study shows that intervention of ginseng maintains the improved pulmonary function of FBC and prevents cardiotoxicity, possibly through the crosstalk of PKA and Epac signaling pathways, inhibition of βAR-Gs-PKA/CaMKII and activation of Epac1/ERK1/2 axis, with subsequent changes in cardiac function and myocardial apoptosis. These results suggest that appropriate drug combination is an effective therapy in cor pulmonale.

## Materials and Methods

### Drugs and Reagents

*Aconiti Lateralis Radix Praeparata* (Fuzi), *Fritillariae Thunbergii bulbus* (Beimu), and *Ginseng Radix et Rhizoma* (Ginseng) were obtained from Chinese herbs Corporation. Ginseng (White Ginseng), Fuzi (Hei Shunpian) Beimu (Zhe Beimu) combination (1:1), and a combination of the three herbs (1:1:1) were soaked for 0.5 h with 10 times distilled water, microboiled for 1 h twice, mixed with the twice filtrate, and then dried at 60°C in a vacuum. The final extractum was stored at -20°C and then diluted with distilled water to the appropriate concentration for further application.

Ginsenoside Re (≥98.0% purity) was purchased from National Institute for Pharmaceutical and Biological Products (Beijing, China), Ginsenosides Rb1, Rg1, and Ro (≥98.0% purity) were purchased from Zhongxin Pharmaceutical Group, Co., Ltd. (Tianjin, China). Other compounds were of analytical grade. Cell Counting Kit-8 (CCK8), creatine kinase (CK), CK-MB and lactate dehydrogenase (LDH) Assay Kit were purchased from Dojindo and Nanjing Jiancheng Bioengineering Institute.

### Experimental Protocols

Male Wistar rats weighing 200–220 g (Certificate No. SCXK 2012-0001) were purchased from Vital River Laboratory Animal Technology, Co., Ltd. (Beijing, China). After a 7-day acclimatization to laboratory conditions, their rats were randomly divided into two groups: control group (Con, *n* = 10, normal saline) and treatment group. The treatment group was composed of rats with pulmonary hypertension induced by a single intraperitoneal injection of monocrotaline in accordance with a preciously described method. After 3 weeks when it comes to late-stage of pulmonary hypertension (cor pulmonale), the rats started to die (two rats died). The remaining rats were randomly divided into four groups (*n* = 26 per group): model group (Mod), ginseng group (Gins, i.g., 5 g/kg/day), FBC (i.g., 5:5 g/kg/day), ginseng combined with FBC (G/FBC, i.g., 5:5:5 g/kg/day). Each drug was administrated for 2 weeks. The ginseng compatibility ratio of 1:1:1 was selected based on clinical usage (Zhang et al., 2010; Yu, 2012). All rats were housed in an environmentally controlled breeding room (temperature: 22 ± 2°C, humidity: 60 ± 5%, 12:12-h dark/light cycles) with *ad libitum* access to water and food. The care and treatment of the animals were performed in accordance with the Guide for the Care and Use of Laboratory Animals, and all experiments were approved by the Ethics Committee for Animal Experimentation of Tianjin University of Traditional Chinese Medicine (TCM-2017-042-E08).

The rat cardiomyoblast cell line H9C2 was maintained in Dulbecco’s modified Eagle’s medium (DMEM, HyClone, United States) containing 10% FBS and 1% penicillin–streptomycin (HyClone, United States) in a 37°C, 5% CO_2_ incubator. Cell medium was changed every 2 days. H9C2 cells were plated at a density of 3 × 10^4^ cells/mL in 96-well plates at 100 μL/well and then treated with Ginsenosides Rb1, Rg1, Re, and Ro (1–200 μmol/L) for 24 h. CCK-8 was used to test the toxicity of the compounds. The cells were seeded at a density of 8 × 10^4^ cells/mL in 6-well plates at a final volume of 2.5 mL per well, and then treated with Ginsenosides Rb1, Re, Rg1, and Ro of appropriate concentration for 24 h. Cell protein was extracted for immunoblotting analysis.

### Echocardiography and Hemodynamic Measurements

After 2 weeks of treatment, echocardiography and hemodynamic parameters were performed in accordance with our previously reported methods (Lu et al., 2015). The left ventricular internal diameter at end-diastole (LVIDd), left ventricular internal diameter at end-systole (LVIDs), left ventricular end-diastolic volume, left ventricular end-systolic volume, right ventricular systolic pressure (RVSP), right ventricular end-diastolic pressure (RVEDP), and mean pulmonary arterial pressure (mPAP) were measured. The left ventricular ejection fraction (EF, %) and the left ventricular fractional shortening (FS, %) were calculated according to CUBED formula.

### Measurement of Serum Myocardial Enzymes

After completion of hemodynamic measurements, blood was collected and centrifuged at 3000 rpm for 15 min at 4°C to separate serum. LDH, CK, and CK-MB were tested by the semi-automatic biochemical analyzer in accordance with the manufacturer’s protocols.

### Tissue Processing and Histopathological Analysis

Rats were euthanized with an overdose of isoflurane (5% isoflurane with 2 L/min oxygen). The viscera (lung, heart, liver, and spleen) were rapidly dissected, and then the heart was separated into the RV and the left ventricle (LV) with interventricular septum (IVS) and then weighed separately. Finally, the viscera weight/body weight and the ratio of the weight of right ventricular wall to the weight of left ventricular wall plus IVS [RV/(LV+IVS)] were measured and calculated as indices of right ventricular hypertrophy.

After weighing, the upper left lung and heart were fixed in 4% buffered paraformaldehyde and underwent hematoxylin/eosin staining. Pulmonary arterial vascular remodeling was blindly measured by the % of wall thickness (total diameter–internal diameter/total diameter) through H&E staining at 200× magnification, with five arteries per lung section. The heart was stained using TUNEL (Roche, Mannheim, Germany) according to the manufacturer’s instructions. Apoptotic cells were counted under a microscope at 400× magnification, and five regions were randomly selected from each section. The apoptosis rate was expressed as the ratio of apoptotic cells to total cells in the same visual field. The RV was divided and stored at -80°C for immunoblotting or placed t in RNAstore reagent (TIANGEN Biotech, Co. Ltd., Beijing, China) for PCR analysis.

### Quantitative Real-Time PCR

Total RNA of myocardial tissue samples in each group was extracted using RNAsimple total RNA kit (Tiangen, Beijing). Total RNA (1 μg) was reversely transcribed into cDNA with FastQuant RT kit (with gDNase) (Tiangen, Beijing) in accordance with the manufacturer’s instructions. The mRNA expression levels for apoptosis-related genes (Bax and Bcl-2) were determined by using SuperReal PreMix Plus (SYBR Green) kit (Tiangen, Beijing) with the CFX96 touch system (Bio-Rad, United States). The quantity of mRNA was normalized for glyceraldehyde-3-phosphate dehydrogenase (GAPDH). Amplification was performed with the following primers:

(1) GAPDH: 5′-GATTTGGCCGTATCGGAC-3′ and 5′-GAAGACGCCAGTAGACTC-3′;
(2) Bax: 5′-CCAAGAAGCTGAGCGAGTGTCTC-3′ and 5′-AGTTGCCGTCTGCAAACATGTCA-3′;
(3) Bcl-2: 5′-AGCTGCACCTGACGCCCTT-3′ and 5′-CAGCCAGGAGAAATCAAACAGAGG-3′.

Amplification conditions were 15 min at 95°C, followed by 40 cycles of 10 s at 95°C and 32 s at 60°C. All PCR reactions were performed in triplicate.

### Immunoblotting

Proteins of heart tissues or cultured cardiomyocytes were obtained using a Protein Extraction Kit (Beyotime Institute of Biotechnology, Inc., Shanghai, China) in accordance with the manufacturer’s protocols. Total protein from the heart tissues or cultured cardiomyocytes was separated by SDS-PAGE gel electrophoresis and transferred to polyvinylidene difluoride membranes (Millipore, United States). The blot was incubated with the following primary antibodies at 4°C over-night, primary antibodies against GADPH (rabbit polyclonal antibody 1:500, Thermo Fisher), β-actin (rabbit polyclonal antibody 1:1000, Abcam), PKA (rabbit polyclonal antibody 1:500, Millipore), GRK2 (rabbit polyclonal antibody 1:500, Abcam), CaMKII (rabbit polyclonal antibody 1:1000, CST), β_2_-AR phosphorylated serine pSer346 and pSer(355,356) (rabbit polyclonal antibody 1:500, Sigma), Epac (Mouse polyclonal antibody 1:1000, CST), p44/42 MAPK(ERK1/2) (rabbit monoclonal antibody 1:1000, CST), and phospho-p44/42 MAPK(ERK1/2) (Thr202/Tyr204) (rabbit monoclonal antibody 1:1000, CST). The membrane was then incubated with the secondary antibody (1:5000; anti-rabbit or anti-mouse IgG conjugated with horseradish peroxidase, ZSGB-BIO ORIGENE, Beijing, China). The relative density of each protein band was analyzed by an imaging densitometer (Cuene Genins). Densitometry values were normalized with GADPH or β-actin.

### Statistical Analysis

Data were presented as mean ± SEM. For comparison of multiple groups, one-way ANOVA followed by the least-significant difference method was applied (SPSS version 19.0, IBM, Inc.). Statistical significance was considered at *P* < 0.05.

## Results

### Ginseng Compatibility Environment Partially Restores Pulmonary Function in MCT-Induced Cor Pulmonale

**FIGURE 1:**
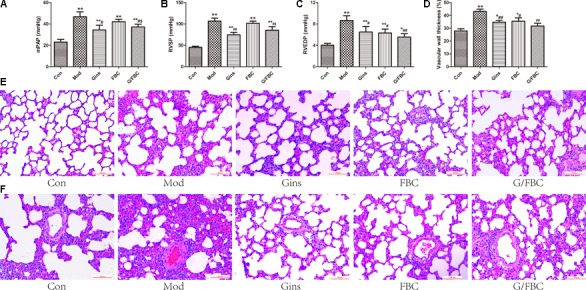
G/FBC improved pulmonary function in MCT-induced cor pulmonale. **(A–C)** Pulmonary parameter and right ventricular function: mPAP, RVSP, and RVEDP. **(D,E)** Representative hematoxylin and eosin staining images in lung tissues, magnification 200×. **(F)** A comparison of the medial thickness of the pulmonary arterial walls in each group. Data are mean ± SEM. ^∗^*P* < 0.05, ^∗∗^*P* < 0.01 vs. Con; ^#^*P* < 0.05, ^##^*P* < 0.01 vs. Mod; ^$^*P* < 0.05, ^$$^*P* < 0.01 vs. FBC. (Con: control group, Mod: model group, Gins: ginseng group, FBC: Fuzi and Beimu compatibility group, G/FBC: ginseng combined with FBC.)

We first evaluated whether the combination of ginseng and Fuzi Beimu could ameliorate pulmonary function in cor pulmonale. Treatment in cor pulmonale rats was initiated 3 weeks after MCT injection, and the pulmonary vascular hemodynamic and histopathological changes were measured after 2 weeks of oral administration. As shown in **Figures 1A–C**, MCT injection resulted in hemodynamic dysfunction, and the mPAP, RVSP, and RVEDP obviously raised. These hemodynamic parameters decreased in the FBC, G/FBC, and ginseng groups, suggesting that pulmonary artery hypertension was alleviated. The mPAP, RVSP, and RVEDP were slightly lower in the G/FBC group compared with the FBC group. Then, we examined the alveolar pathological changes and the extent of pulmonary vascular remodeling as a surrogate for lung injury and measured the medial thickness of the pulmonary arterioles (**Figures 1D–F**). The alveolar septum widened and inflammatory cells were infiltrated obviously. Lumen stricture or even obliteration was observed, and the medial wall thickness of pulmonary arterioles markedly increased in the model rats. The pulmonary pathological changes and pulmonary vascular remodeling were similarly ameliorated in the FBC, G/FBC, and Gins group. These results suggest that ginseng compatibility environment can ameliorate pulmonary function even slightly better than FBC can. Meanwhile, the ginseng group also showed effect.

### Ginseng Compatibility Environment Attenuates Cardiotoxicity Triggered by FBC Treatment

**FIGURE 2:**
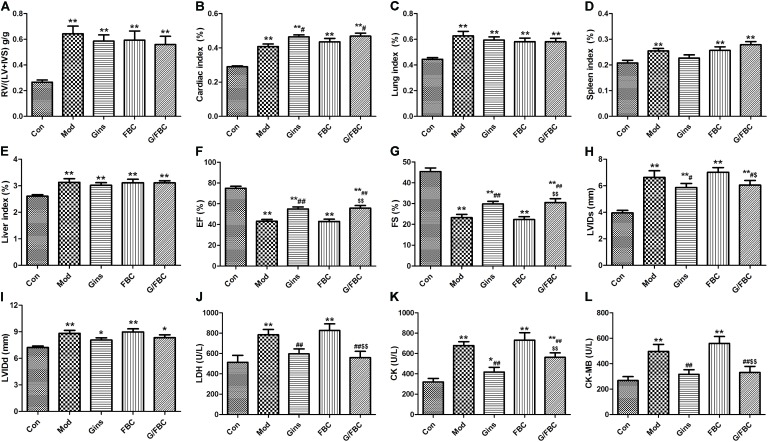
Fuzi and Beimu compatibility-induced cardiotoxicity in cor pulmonale were attenuated by ginseng intervention. **(A–E)** Effects of ginseng compatibility on viscerosomatic index: RV/(LV+IVS), cardiac, lung, spleen, and liver index. **(F–I)** Indicators of cardiac function: EF, FS, LVIDs, and LVIDd. **(J–L)** Serum myocardial enzymes: LDH, CK, and CK-MB. Data are presented as mean ± SEM. ^∗^*P* < 0.05, ^∗∗^*P* < 0.01 vs. Con; ^#^*P* < 0.05, ^##^*P* < 0.01 vs. Mod; ^$^*P* < 0.05, ^$$^*P* < 0.01 vs. FBC.

To assess the potential impact of ginseng compatibility environment on cardiotoxicity changes to cor pulmonale, we determined the changes in the viscera index, cardiac function, and myocardial enzymes (**Figure 2**). The MCT-treated rats showed significant increases in the right ventricular hypertrophy and in the cardiac, lung, spleen, and liver indexes (**Figures 2A–E**). A slight difference in the viscera index was found between the FBC and model groups. Nevertheless, compared with the FBC group, the G/FBC group had a tendency to decrease in right ventricular hypertrophy and increase in cardiac and spleen indexes. This result indicates that myocardial hypertrophy and immune function enhancement.

MCT-injection resulted in cardiac dysfunction, EF and FS significantly reduced, LVIDs and LVIDd obviously raised (**Figures 2F–I**). These indications in the FBC were similar with the model group. While G/FBC prevented the massive changes of cardiac function seen after FBC treated. And ginseng group can also prevent the massive alterations compared to model group.

We further investigated the production of specific cardiac damage markers, such as serum LDH, CK, and CK-MB. We observed significant accumulations of myocardial enzymes in the model group (**Figures 2J–L**). In analogy, the expression levels of myocardial enzymes were higher in the FBC group compared with the model group. By contrast, G/FBC obviously decreased myocardial enzymes as compared with FBC. Meanwhile, the myocardial enzymes significantly decreased in the ginseng group compared with the model group. Collectively, these data suggest that ginseng compatibility environment can attenuate the cardiotoxicity induced by Fuzi Beimu incompatibility intervening in cor pulmonale.

### Ginseng Compatibility Environment Alleviates Cardiac Pathological Changes and Myocardial Apoptosis

**FIGURE 3:**
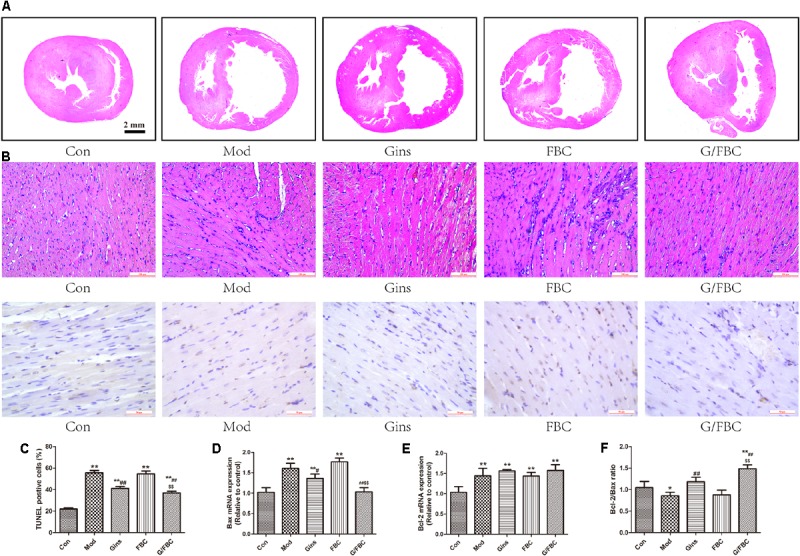
Impaired cardiac histomorphology and myocardial apoptosis were ameliorated by ginseng compatibility environment. **(A)** The change of morphology of transversal septal sections of hearts and right ventricle (RV), magnification 200×. **(B,C)** Myocardial apoptosis: DNA fragmentation of apoptotic cells was detected by TUNEL staining in myocardium and the apoptosis rates in different groups were calculated, magnification 400×. **(D–F)** Apoptosis-related mRNA: Bax, Bcl-2, and the ration of Bcl-2 to Bax in RVs. The values represent means ± SEM. ^∗^*P* < 0.05, ^∗∗^*P* < 0.01 vs. Con; ^#^*P* < 0.05, ^##^*P* < 0.01 vs. Mod; ^$^*P* < 0.05, ^$$^*P* < 0.01 vs. FBC.

We next investigated how ginseng compatibility environment-induced cardiotoxicity attenuation is paralleled by improvement of histopathology. MCT resulted in right ventricular impairment, as demonstrated by RV dilation, cardiomyocyte disorder, unclear muscle fiber direction, dissolution of myocardial cell sarcoplasm to increase interstitial fluid, and inflammatory cell infiltration (**Figure 3A**). Small difference in right ventricular histopathology was found between the model and FBC groups, but inflammatory cell infiltration was more obvious in the latter than in the former. These results suggest that the addition of ginseng prevented the progressive increase in RV dilation and histopathological changes induced by FBC. Moreover, the ginseng group exerted a cardioprotective effect to reduce RV dilation, myocardial fibrosis, and inflammatory cell infiltration as compared with model group.

Cardiomyocyte apoptosis was identified and calculated using TUNEL staining (**Figures 3B,C**). As expected, MCT exposure significantly induced of cardiomyocyte apoptosis. No significant difference in apoptosis was found between the model and FBC groups, but the G/FBC group showed markedly lower apoptosis rate compared with the FBC group. Compared with the model group, the ginseng group had significantly lower apoptosis rate. We then examined the expression of apoptosis-related genes Bax, Bcl-2 and the ratio of Bcl-2 to Bax (**Figures 3D–F**). In line with the results of TUNEL staining, G/FBC treatment notably suppressed Bax expression and increased Bcl-2/Bax ratio when compared with FBC. The expression levels of Bax and Bcl-2/Bax were similarly affected by ginseng treatment alone.

### Coordinated Crosstalk of PKA and Epac Signaling Contributes to the Toxicity-Attenuated Effect of Ginseng Compatibility Environment

**FIGURE 4:**
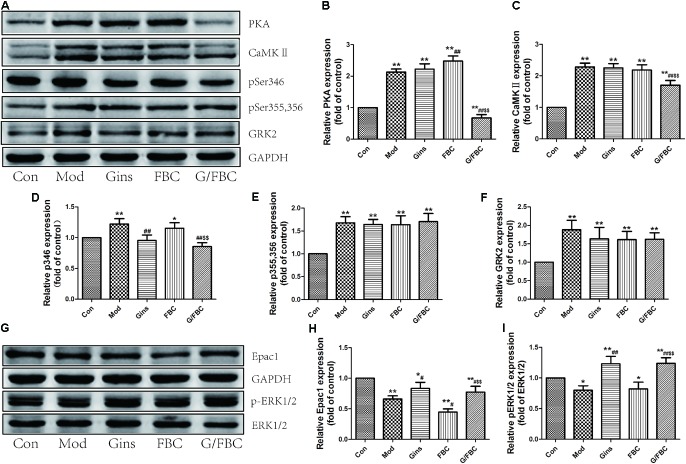
Ginseng compatibility environment-induced toxicity-attenuated effect was a result of crosstalk of inhibition of βAR-Gs-PKA/CaMKII and activation of Epac1/ERK1/2 signaling axis. **(A–F)** Protein levels of PKA, CaMKII, pSer346-β2AR, pSer355, 356-β2AR, and GRK2 were analyzed. **(G–I)** Epac1 and the ratio of pERK1/2 to ERK1/2 were detected. The blots were quantified and presented as the fold of control levels. The values represent means ± SEM. ^∗^*P* < 0.05, ^∗∗^*P* < 0.01 vs. Con; ^#^*P* < 0.05, ^##^*P* < 0.01 vs. Mod; $*P* < 0.05, $$*P* < 0.01 vs. FBC.

The βAR-Gs-PKA/CaMKII signaling pathway plays an important role in FBC-induced cardiotoxicity (Zhuang et al., 2016), and the Epac1/ERK1/2 signaling axis reveals a protective role in the heart (Zhang et al., 2013; Mangmool et al., 2015). In the present study, we assessed the alteration of these signaling pathways in MCT-induced cor pulmonale after treatment with ginseng compatibility (**Figure 4**). Our results showed that the protein levels of PKA, CaMKII, pSer346-β_2_AR, pSer355,356-β_2_AR, and GRK2 were significantly up-regulated in model group (**Figures 4A–F**). This result is consistent with our previous study. PKA protein expression was largely increased in FBC, and no significant difference in the expression of other proteins was found between the FBC-treated and model groups. Nevertheless, G/FBC treatment obviously down-regulated the expression of PKA, CaMKII and pSer346-β_2_AR, but did not alter the protein levels of pSer355,356-β_2_AR and GRK2. Ginseng treatment alone did not significantly change the proteins levels of PKA, CaMKII, pSer355,356-β_2_AR, and GRK2, but down-regulated that of pSer346-β_2_AR compared with the model group. We then tested the Epac signaling, which was independent from PKA signaling (**Figures 4G–I**). The protein levels of Epac1 and the ratio of pERK1/2 to ERK1/2 were markedly reduced in the model group. And in the FBC-treated group, Epac1 protein expression was further reduced, and the ratio of pERK1/2 to ERK1/2 showed no significant change compared with the model group. Significant differences were found in the G/FBC group, and Epac1 and ratio of pERK1/2 to ERK1/2 were markedly enhanced compared with those in the FBC group. Similar changes were also observed in the ginseng group when compared with the model group. In sum, these findings indicate that the inhibition of the βAR-Gs-PKA/CaMKII signaling pathway but not GRK2-dependent β_2_AR-Gi signaling and activation of the Epac1/ERK1/2 axis in cardiac function is associated with the capacity of ginseng compatibility environment to attenuate toxicity and maintain efficacy.

## Discussion

The present study is the first to disclose that ginseng compatibility environment plays a crucial role in treating cor pulmonale. It partially restored lung function and significantly inhibited cardiotoxicity, which elucidated that the drug combination has a unique advantage in the treatment of chronic and complex diseases. For cancer treatment, the use of ginseng and its extracts as adjuvant to improve curative effects has been reported in both animal models and clinical applications (Zhang et al., 2017; Zhao et al., 2017).

β_2_-ARs agonists, as the most potent and prevalent bronchodilators, have been used for the treatment of lung diseases, such as COPD and asthma (Fang et al., 2015; Monaco and Hanania, 2017). β_2_-ARs that are coupled via a stimulatory G protein to adenylyl cyclase increase cAMP accumulation. We previously demonstrated that the main chemicals of Beimu could suppress the PDK1/Akt/PDE4D axis, which physiologically controls cAMP hydrolysis, and then increase cAMP accumulation. Moreover, some alkaloids of Fuzi can activate β_2_-ARs (Yang et al., 2015; Zhuang et al., 2016). FBC serves a synergistic function on β_2_-ARs activation, and FBC can ameliorate lung function and alleviate pulmonary pathological changes in early-stage of pulmonary hypertension. Our data showed that G/FBC also improved pulmonary function, even slightly better than FBC did. The restored pulmonary function is supported by findings that ginsenosides can suppress the contractile response of pulmonary arteries and attenuate pulmonary hypertension, the mechanism maybe via NO and MAPK pathways (Wang R.X. et al., 2015; Qin et al., 2016).

Although constant β-ARs activation may facilitate the development of heart failure, β-adrenergic signaling in heart failure exerts both cardiotoxic and cardioprotective effects through the PKA and Epac dual pathways (Kwak et al., 2008; Zhang et al., 2013; Wang Y. et al., 2015). Epac, a newly identified cAMP signaling target that is independent from PKA, also plays critical roles in the regulation of various cAMP-dependent cardiovascular functions, such as calcium handling, vascular tone, hypertrophy, and apoptosis (Wu et al., 2014; Laurent et al., 2015; Lezoualc’h et al., 2016). PKA-dependent activation of Ca/CaMKII contributes to cardiomyocyte death, whereas, Epac-dependent ERK signaling activation may protect cardiac myocytes from death. These pieces of evidence have shown that the balance of PKA and Epac signaling pathways for the treatment of several cardiovascular disorders is worthy of further studies.

**FIGURE 5:**
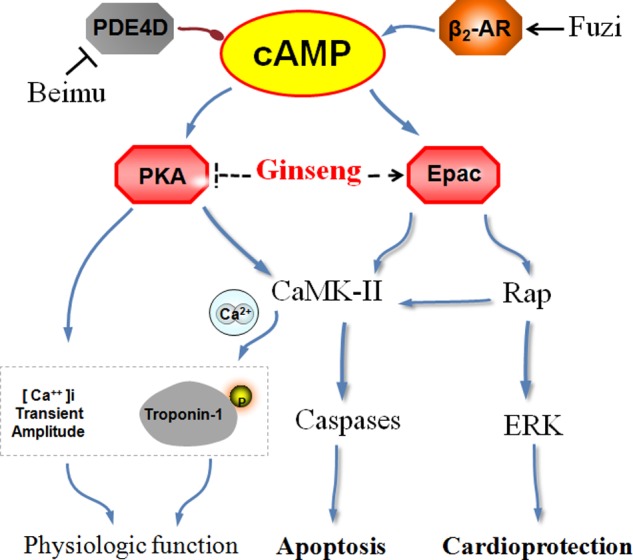
A schematic for ginseng compatibility environment mediated cardiotoxicity-attenuated effect by the crosstalk of PKA and Epac signaling axis. Previous study had shown that component in Beimu inhibited PDE4D axis and component in Fuzi activated β_2_AR. And FBC aggravated the heart injury through a synergistic activation of β_2_AR signaling pathway. This study identifies that ginseng via inhibiting βAR-Gs-PKA/CaMKII signaling axis, and activating the Epac1/ERK1/2 signaling pathway, resulted in the toxicity-attenuated effect in cor pulmonale.

**FIGURE 6:**
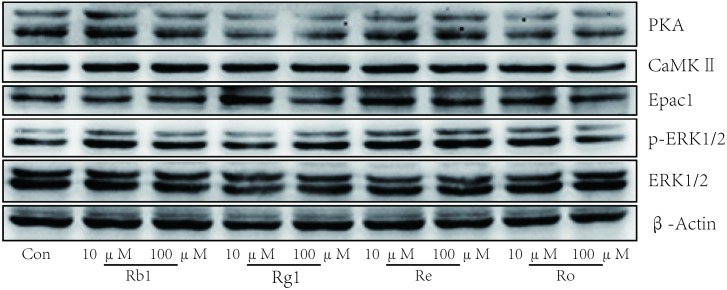
Ginsenosides played critical roles in the effect of toxicity attenuated of ginseng compatibility. Protein levels of PKA, CaMKII, Epac1, ERK1/2, and pERK1/2 were tested after ginsenoside Rb1, Rg1, Re, Ro treated in H9C2 cells.

Ginseng compatibility environment protects against cardiomyocyte apoptosis possibly through the coordinated crosstalk of PKA and Epac signaling pathways (**Figure 5**). The difference between the control and FBC groups in the protein levels of PKA, CaMKII, and pSer346-β2AR was largely abolished after treatment with ginseng compatibility environment. Moreover, the down-regulation of Epac1 and the ratio of pERK1/2 to ERK1/2 were restored in the G/FBC group. Ginsenosides played critical roles in the toxicity attenuation of ginseng compatibility environment (**Figure 6**). Ginsenosides Rb1, Rg1, Re, and Ro, which are representative components of ginseng, all influenced the protein expression of PKA, CaMKII, Epac1, and pERK1/2. In agreement with our results, other publications have reported total ginsenosides exert a cardioprotective effect via the ERK signaling pathway (Li et al., 2013; Qin and Wei, 2015).

## Conclusion

Ginseng combined with Fuzi Beimu ameliorated pulmonary function and attenuated cardiotoxicity induced by FBC in cor pulmonale, which is at least partly mediated by the crosstalk of the βAR-Gs-PKA/CaMKII and Epac1/ERK1/2 signaling pathways. Ginseng compatibility environment would be an effective therapy in cor pulmonale. The coordinated crosstalk of the PKA and Epac signaling pathways may be an attractive therapeutic target for the treatment of cardiovascular diseases. Taken together, our study revealed new pharmacological targets and provided a novel strategy to treat cor pulmonale.

## Author Contributions

PZ and YZ designed study. YH was a major contributor in writing the manuscript and performing the experiments. XL, LL, and SF analyzed the data and perform the experiments. All authors read and approved the final version of the manuscript.

## Conflict of Interest Statement

The authors declare that the research was conducted in the absence of any commercial or financial relationships that could be construed as a potential conflict of interest.
